# A new method for measuring torsional deformity in scoliosis

**DOI:** 10.1186/1748-7161-6-7

**Published:** 2011-04-16

**Authors:** Toshio Doi, Satoshi Kido, Umito Kuwashima, Osamu Tono, Kiyoshi Tarukado, Katsumi Harimaya, Yoshihiro Matsumoto, Kenichi Kawaguchi, Yukihide Iwamoto

**Affiliations:** 1 Department of Orthopaedic Surgery, Kyushu University Beppu Hospital, Oita, Japan; 2 Department of Orthopaedic Surgery, Graduate School of Medical Sciences, Kyushu University, Fukuoka, Japan

## Abstract

**Background:**

The importance of spinal rotational and torsional deformity in the etiology and the management of scoliosis are well-recognized. For measuring the posterior spinal component rotation, Ho's method was reported to be reliable. However, there is no practical method to measure the anterior spinal component rotation. Moreover, there is also no method to quantify the spinal torsional deformity in scoliosis. The goal of this study is to characterize scoliosis and its deformity to hypothesize the etiology and the development of scoliosis, and to establish a new method for the measurement of the vertebral body rotation and spinal torsional deformity in scoliosis using CT scans.

**Methods:**

Pre-operative CT scans of 25 non-congenital scoliosis patients were recruited and the apical vertebral rotation was measured by a newly developed method and Ho's method. Ho's method adopts the laminae as the rotational landmark. For a new method to measure the apical vertebral rotation, the posterior point just beneath each pedicle was used as a landmark. For quantifying the spinal torsional deformity angle, the rotational angle difference between the two methods was calculated.

**Results:**

Intraobserver and interobserver reliability analyses showed both methods to be reliable. Apical vertebral rotation revealed 13.9 ± 6.8 (mean ± standard deviation) degrees by the new method and 7.9 ± 6.3 by Ho's method. Right spinal rotation was assigned a positive value. The discrepancy of rotation (6.1 ± 3.9 degrees), meaning that the anterior component rotated more than the posterior component, was considered to express the spinal torsional deformity to the convex side.

**Conclusions:**

We have developed an easy, reliable and practical method to measure the rotation of the spinal anterior component using a CT scan. Furthermore, we quantified the spinal torsional deformity to the convex side in scoliosis by comparing the rotation between the anterior and posterior components.

## Background

Besides coronal side curvature, axial deformities are essential in structural scoliosis. Although the rotational deformity has been well-described [1-8], little is known about torsional deformity in scoliosis. Based on observations of 3D images or specimens of scoliosis, the anterior component, such as the vertebral body, has thus been determined to rotate more than the posterior component, such as the spinous process and laminae [9,10]. Clarifying the rotation and the torsion is thus considered to be important for understanding the etiology and for achieving the better management of scoliosis.

Various methods have been developed and used to measure the vertebral rotation in scoliosis. For example, Nash and Moe published a practical method for determing the vertebral rotation from the projection of the pedicle in frontal radiographs [11]. The Perdriolle torsion meter is a template used to measure the amount of vertebral rotation on spinal radiographs, measuring the position of the pedicles with regard to the vertebral body on radiographs [12]. Computed tomography (CT) scan measurement is a good tool for axial plane deformity evaluation in scoliosis [1-8]. Aaro and Dahlborn introduced the use of CT scans to measure vertebral rotation [7,8], and Ecker et al. used the sagittal angle, which was taken to be the angle between the sagittal plane and the line between the correct posterior mid-point of the vertebra canal and the center point of the corpus vertebrae, to determine the rotation [6]. Krismer et al. described a new method to measure aixial rotation using several anatomical points on CT scans [1]. Gocen used the connection line of the most posterior points of two pedicles [5], and Kouwenhoven et al. used the gravity center calculated using an automatic computer program for the rotational measurement [2]. In contrast, Ho's method uses the laminae as the landmark [3,4]. However, although these numerous methods have been proposed for the vertebral rotation, it is difficult to find an anatomical landmark to measure the anterior component rotation [2,13], and few practical methods are available.

Presently, we herein report on the development of a new method to measure the spinal anterior component rotation. Furthermore, the spinal torsional deformity in scoliosis was quantified by a combination of our new method and Ho's method.

## Methods

### Patients and CT scan imaging

CT scans of 25 patients, aged 12-20-years-of-age (mean 15.0 years), treated in our institution from 2008-2010 for thoracic scoliosis were retrospectively reviewed. Congenital scoliosis was eliminated in this series. All had structural right thoracic curvature. Cobb angle of the thoracic curve, measured in standing AP position using a long cassette, was 56.5 (33-77 degrees). A CT scan was performed to assess the spinal deformity and pedicle size for the spinal instrumentation. No new CT scan examination was ordered for the current study. All CT images were measured on the computer screen by the measurer (Fuji Synapse System, Japan). The present project received approval from the Ethical Commission of our institute. Written informed consent was obtained from the patient for publication of accompanying images. A copy of the written consent is available for review by the Editor-in-Chief of this journal.

### Rotation measurement by Ho's method

**Figure 1:**
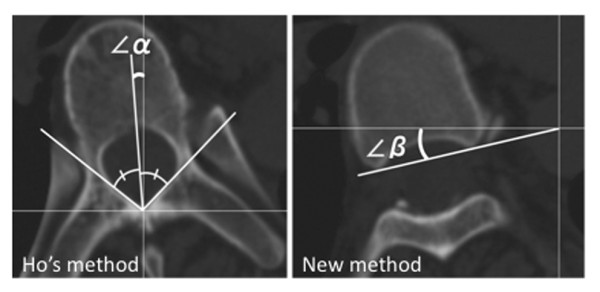
**CT measurement methods**. Measurement of spinal rotation by Ho's method and by the new method are shown. The inner lamina surface is the anatomical landmark of Ho's method (angle α). The posterior vertebral body beneath the pedicle is the anatomical landmark for the new method (angle β).

The apical vertebra was defined as the most laterally deviated vertebra or disc in a scoliosis curve in the global axis system using standing AP radiographs [14] and the apical vertebra was chosen for the CT measurement. In patients whose apical vertebrae were at the disc level, the upper adjacent vertebrae were first chosen for the CT measurement. In patients in whom both pedicles of the apical vertebra could not be seen in one CT slice, we measured the next vertebra that was parallel to the CT gantry, as it is otherwise difficult to apply our method. In Ho's method, a line bisecting the angle formed by the two laminae was drawn on computer screen, and the angle of vertebral rotation was taken as the angle between this line and the vertical plane [4] (Figure 1., ∠α). The right spinal rotation angle was assigned a positive value.

### Rotation measurement by the new method

In the new method, the posterior point of vertebral body just beneath each pedicle was marked. The line was drawn joining these two points on the computer screen. The angle of vertebral rotation was taken as the angle between this line and the horizontal plane (Figure 1., ∠β). The right spinal rotation angle was assigned a positive value. On CT images, both methods were applied to the same vertebrae.

### Torsional deformity angle

The torsional deformity angle was defined as the rotational discrepancy of the two methods (∠α∠β). The right spinal torsional angle was given a positive value.

#### Reliability analysis

Three orthopaedic surgeons (observer 1, observer 2, and observer 3) were familiarized with the computer program and also taught how to place the vertebral landmarks on the computer monitor. The measurements were carried out twice on different occasions with 25 scoliosis patients. The intervals between measurements were at least 2 weeks. Intraobserver and interobserver agreement was assessed by the interclass correlation coefficient.

#### Statistical analysis

Pearson correlation was applied for the correlation of torsional deformity angle to Cobb angle or rotational deformity. Student's *t-*test was applied for the comparison of rotation angle using the new and Ho's methods. A *p *value of 0.05 was considered to be significant.

## Results

**Figure 2:**
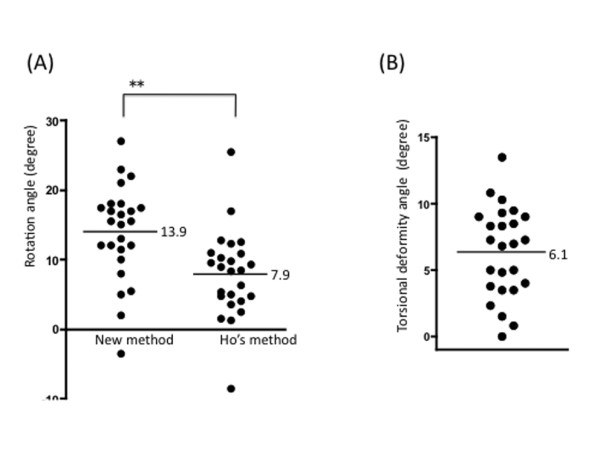
**Anterior component rotated more than posterior component**. The rotation angle measured by the new method (13.9 ± 6.8 (mean ± SD) degrees) is significantly larger than the rotation angle measured by Ho's method (7.9 ± 6.3 degrees). (B) This discrepancy of rotation between anterior and posterior components (6.1 ± 3.9 degrees) represents the spinal torsional deformity. **p < 0.001

The apical vertebrae were from T7 to T10.5 on a standing AP radiograph. In two patients for whom both pedicles could not be seen in one CT slice, the upper adjacent vertebra was select for measurement. CT scan values of rotation were 13.9 ± 6.8 (mean ± standard deviation) in the new method and 7.9 ± 6.3 in Ho's method (Figure 2A).

The interclass correlation coefficient for the intraobserver reliability of the measurements using the new method was 0.982 for observer 1, 0.977 for observer 2, and 0.977 for observer 3 (Table 1). The interclass correlation coefficient for the intraobserver reliability of the measurements using Ho's method was 0.966 for observer 1, 0.902 for observer 2, and 0.971 for observer 3.

**Table 1:** Intraobserver reliability analysis for a new method and Ho's method

Observer	New method	Ho's method
Observer 1 (N = 25)	0.98 (0.97-0.99)	0.97 (0.94-0.98)

Observer 2 (N = 25)	0.98 (0.96-0.99)	0.90 (0.82-0.95)

Observer 3 (N = 25)	0.98 (0.96-0.99)	0.97 (0.95-0.99)

Each observer measured CT images (N = 25) and interclass correlation coefficient was calculated. Numerical value shows the gamma of interclass correlation coefficient and the range of the 95% confidence interval.

The interclass correlation coefficient for the interobserver reliability of the measurements by the new method and by Ho's method by the three observers was 0.890 and 0.782 respectively (Table 2).

**Table 2:** Interobserver reliability analysis for a new method and Ho's method

	Interclass correlation coefficient	95% confidence interval
New method	0.89	0.81 - 0.94

Ho's method	0.78	0.65 - 0.88

Each observer measured CT images (N = 25) twice on different occasions and the interclass correlation coefficient was calculated.

To quantify the spinal torsional deformity, the difference between the anterior and posterior components was calculated. The new method, which uses the posterior part of vertebral body, is considered to express the spinal anterior component rotation. Ho's method, which uses the inner surface of the laminae, is considered to express the spinal posterior component. The anterior component rotation was significantly larger than posterior component rotation (Student's *t-*test: *p *= 0.002). The discrepancy of rotation between the posterior and anterior components was 6.1 ± 3.9 degrees, which is considered to express the spinal torsional deformity to the convex side (Figure 2B).

**Figure 3:**
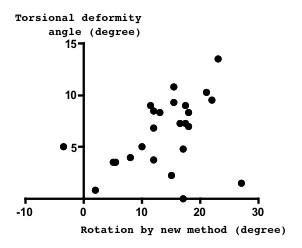
**Relation between spinal anterior component rotation and torsional deformity**. Spinal rotation measured by the new method, which is supposed to express the anterior component rotation, seemed to correlate with the spinal torsional deformity angle (*r *= 0.38, *p *= 0.060).

**Figure 4:**
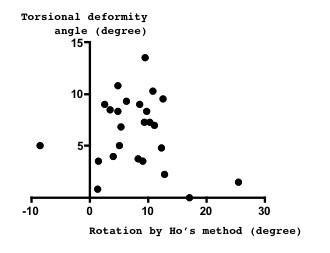
**Relation between spinal posterior component rotation and torsional deformity**. Spinal rotation measured by Ho's method, which expresses the posterior component rotation, did not correlate closely with the torsional deformity angle (*r *= - 0.17, *p *= 0.429).

Spinal torsional deformity angle seemed to correlate with the rotation angle measured by the new method (*r *= 0.38, *p *= 0.060) (Figure 3), but not as measured by Ho's method (*r *= -0.17, *p *= 0.429) (Figure 4).

**Figure 5:**
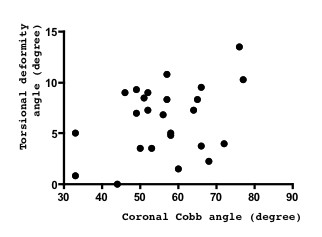
**Relation between coronal curvature and spinal torsional vertebral deformity**. The coronal Cobb angle appears to correlate with the spinal torsional angle (*r *= 0.36, *p *= 0.077).

The relation between the coronal Cobb angle and torsional deformity was analyzed (Figure 5). Coronal Cobb angle and torsional vertebral deformity angle seemed to correlate but was not significant (*r *= 0.36, *p *= 0.077).

## Discussion

Spinal rotation exists in structural scoliosis and is clearly observed by CT scans. Lamina [3], spinous process, accessory process [5], and vertebral body [2,8] are used as the landmarks for rotational measurement on CT scans. However, because of the spinal body deformity it is sometimes difficult to define the anatomical structures. Ho's method, which is defined by bisecting the bilateral laminae angle, is reported to be useful. As the lamina is an anatomical landmark for Ho's method, it expresses the rotation of spinal posterior component. For measuring the spinal anterior component rotation such as the vertebral body itself, it is difficult to define the rotational angle because of the deformity [10,13] and the lack of any anatomical points.

The apical vertebrae are most laterally deviated in the coronal plane and are usually not tilted. Horizontal vertebrae with the CT scanning of each pedicle can be chosen in all patients. In our series, both pedicles could not be seen in one CT slice in two patients. The differences of the measuring posture (standing for the AP radiograph and supine for the CT scan) are supposed to represent the discrepancy of horizontal vertebrae. Presently, we applied a new method that uses the posterior part of the vertebral body just beneath each pedicle. Compared with Ho's method, our new method was easier and more practical in daily medical practice. Furthermore, our new method was confirmed to be reliable by both intraobserver and interobserver analyses.

There are several important considerations that should be kept in mind when using our new measurement method. One is that our new method can only be applied to the vertebra where both pedicles can be seen in the CT slice. As the CT gantry is vertical to the floor, patient position critically influences the axial CT slice. In the measurement of scoliosis patients, our method can be applied only to the vertebrae around the apex. Another factor that should be kept in mind is that the rotation determined using our new method is a relative value. In this study, as we subtracted the value using two different measuring methods, we did not normalize the rotation angle. When the vertebral rotation is measured using our new method, and the values are compared for different patients, normalization should be done using any anatomical point, such as the sacral or iliac points.

The apical vertebral body adopts a complicated deformity in scoliosis. The spinal anterior component has been confirmed to be rotated more than posterior component by the observation of 3D images or by the specimen findings [9,10]. The deformity of the apical vertebra in the transverse plane consists of a gradual torsion between the posterior complex and the vertebral body. The vertebral body is maximally rotated towards the convexity of the scoliotic curve, whereas the tip of the spinous process is pointed to the posterior. However, precisely why such a spinal torsional deformity exists and also why no quantitative measurement method has yet been established together remain unclear. Furthermore, the anterior vertebral body shape may change by the additional bone formation on the opposite side of the rotational deformity during growth [13,15] in scoliosis. The complicated deformity makes it difficult to locate the anatomical landmarks of the anterior component. As a result, it is difficult to measure the anterior component rotation and to measure the spinal torsional deformity. We presently used the clear anatomical landmark of the posterior border of the vertebral body, which seemed to have less deformity by bony remodeling compared to more anterior vertebral body. By the combined use of our new method (expressing the spinal anterior component rotation) and Ho's method (expressing the spinal posterior component rotation), we could quantify the rotational discrepancy between the anterior and posterior, which is considered to represent the torsional deformity. The method described here is influenced by many factors, including the shape of the posterior part of the vertebral body, the pedicle length, and the lamina shape. Our method can measure the apical vertebral intrinsic torsion, but not the torsion of the vertebral body itself, nor the vertebral torsion in the global spinal system.

We clarified that the anterior component rotated more than posterior component. As the etiology of scoliosis is not known, the order at which such a deformity first occurs is not known. The existence of torsional deformity, the spinal anterior component rotated more than the posterior component, evokes the hypothesis that spinal anterior compartment deformities occur earlier than those of the posterior compartment in the worsening of scoliosis. Although not statistically significant, the present finding that torsional deformity seemed to correlate with anterior component rotation (Figure 3), but not with the posterior component (Figure 4), support the view that spinal anterior component is critical for the etiology of scoliosis deformity. According to our observations, the coronal curvature seemed to correlate with the spinal torsional deformity, but this correlation was not significant (Figure 5). To clarify the mechanisms of deformity, further investigation of the early change of such deformity in scoliosis is needed.

Right thoracic scoliosis, trunk asymmetry and thoracic vertebral right rotation are among the characteristics of adolescent idiopathic scoliosis. Even in the normal spine, trunk asymmetry [16-18], thoracic vertebral right rotation [2] and right thoracic curvature [19-21] have been reported. We were interested in whether torsional deformity exists in the normal spine, and we measured the vertebral rotation using both the new method and Ho's method at the T8 level. Interestingly, the discrepancy of the rotation was 2.6 ± 1.5 degrees (n = 25, adult females), which was considered to indicate the presence of spinal torsional deformity to the right side, even in the normal spine, at the T8 level (*p *= 0.0063). These deformities, which are the same features seen in scoliosis patients, support the possibility that the worsening of deformities existing in normal individuals may underlie the development and progression of scoliosis.

Clinically, paying attention to the difference between the spinal posterior and anterior components is important. During posterior scoliosis surgery, only the posterior component is visible. To avoid an overestimation of the rotational correction, the surgeon has to recognize the existence of such torsional deformity; in other words, understand that the anterior spinal component rotates more than the posterior component.

## Conclusions

We have developed an easy, reliable and practical method to measure the rotation of the spinal anterior component using a CT scan. Furthermore, we quantified the spinal torsional deformity to the convex side in scoliosis by comparing the rotation between the anterior and posterior components.

## Competing interests

The authors declare that they have no competing interests.

## Authors' contributions

TD has contributed to conception and design of the study, acquisition of data, analysis and interpretation of data, and drafting the manuscript. SK, UK, OT and KT performed part of acquisition of data. KH, YM and KK performed part of literature review. YI participated in design and coordination and helped to draft the manuscript. All authors read and approved the final manuscript.

## References

[B1] Krismer M Sterzinger W Haid C Frischhut B Bauer R Axial rotation measurement of scoliotic vertebrae by means of computed tomography scans Spine (Phila Pa 1976) 1996 21 576 581 8852312 10.1097/00007632-199603010-00009

[B2] Kouwenhoven JW Vincken KL Bartels LW Castelein RM Analysis of preexistent vertebral rotation in the normal spine Spine 2006 31 1467 1472 10.1097/01.brs.0000219938.14686.b3 16741456

[B3] Ho EK Upadhyay SS Ferris L Chan FL Bacon-Shone J Hsu LC Leong JC A comparative study of computed tomographic and plain radiographic methods to measure vertebral rotation in adolescent idiopathic scoliosis Spine (Phila Pa 1976) 1992 17 771 774 1502641 10.1097/00007632-199207000-00008

[B4] Ho EK Upadhyay SS Chan FL Hsu LC Leong JC New methods of measuring vertebral rotation from computed tomographic scans. An intraobserver and interobserver study on girls with scoliosis Spine (Phila Pa 1976) 1993 18 1173 1177 8362322 10.1097/00007632-199307000-00008

[B5] Gocen S Havitcioglu H Alici E A new method to measure vertebral rotation from CT scans Eur Spine J 1999 8 261 265 10.1007/s005860050170 10483826 PMC3611174

[B6] Ecker ML Betz RR Trent PS Mahboubi S Mesgarzadeh M Bonakdapour A Drummond DS Clancy M Computer tomography evaluation of Cotrel-Dubousset instrumentation in idiopathic scoliosis Spine (Phila Pa 1976) 1988 13 1141 1144 3206272 10.1097/00007632-198810000-00015

[B7] Aaro S Dahlborn M The longitudinal axis rotation of the apical vertebra, the vertebral, spinal, and rib cage deformity in idiopathic scoliosis studied by computer tomography Spine (Phila Pa 1976) 1981 6 567 572 7336279 10.1097/00007632-198111000-00007

[B8] Aaro S Dahlborn M Estimation of vertebral rotation and the spinal and rib cage deformity in scoliosis by computer tomography Spine (Phila Pa 1976) 1981 6 460 467 7302680 10.1097/00007632-198109000-00007

[B9] Porter RW Idiopathic scoliosis: the relation between the vertebral canal and the vertebral bodies Spine (Phila Pa 1976) 2000 25 1360 1366 10828917 10.1097/00007632-200006010-00007

[B10] Wever DJ Veldhuizen AG Klein JP Webb PJ Nijenbanning G Cool JC v Horn JR A biomechanical analysis of the vertebral and rib deformities in structural scoliosis Eur Spine J 1999 8 252 260 10.1007/s005860050169 10483825 PMC3611175

[B11] Nash CL Jr Moe JH A study of vertebral rotation J Bone Joint Surg Am 1969 51 223 229 5767315

[B12] Perdriolle R Vidal J Thoracic idiopathic scoliosis curve evolution and prognosis Spine (Phila Pa 1976) 1985 10 785 791 4089651 10.1097/00007632-198511000-00001

[B13] Smith RM Pool RD Butt WP Dickson RA The transverse plane deformity of structural scoliosis Spine (Phila Pa 1976) 1991 16 1126 1129 1948404 10.1097/00007632-199109000-00020

[B14] Stokes IA Three-dimensional terminology of spinal deformity. A report presented to the Scoliosis Research Society by the Scoliosis Research Society Working Group on 3-D terminology of spinal deformity Spine (Phila Pa 1976) 1994 19 236 248 8153835

[B15] Kotwicki T Napiontek M Torsional deformity of apical vertebra in adolescent idiopathic scoliosis Stud Health Technol Inform 2002 88 360 364 15456062

[B16] Burwell RG James NJ Johnson F Webb JK Wilson YG Standardised trunk asymmetry scores. A study of back contour in healthy school children J Bone Joint Surg Br 1983 65 452 463 6874719 10.1302/0301-620X.65B4.6874719

[B17] Vercauteren M Van Beneden M Verplaetse R Croene P Uyttendaele D Verdonk R Trunk asymmetries in a Belgian school population Spine (Phila Pa 1976) 1982 7 555 562 7167828 10.1097/00007632-198211000-00008

[B18] Grivas TB Burwell GR Vasiliadis ES Webb JK A segmental radiological study of the spine and rib--cage in children with progressive infantile idiopathic scoliosis Scoliosis 2006 1 17 10.1186/1748-7161-1-17 17049098 PMC1635062

[B19] Taylor JR Vascular causes of vertebral asymmetry and the laterality of scoliosis Med J Aust 1986 144 533 535 3520254 10.5694/j.1326-5377.1986.tb112281.x

[B20] Goldberg C Dowling FE Handedness and scoliosis convexity: a reappraisal Spine (Phila Pa 1976) 1990 15 61 64 2326712 10.1097/00007632-199002000-00001

[B21] Doi T Harimaya K Mitsuyasu H Matsumoto Y Masuda K Kobayakawa K Iwamoto Y Right thoracic curvature in the normal spine J Orthop Surg Res 2011 6 4 10.1186/1749-799X-6-4 21232160 PMC3032747

